# Controlled Growth of Semiconducting ZnO Nanorods for Piezoelectric Energy Harvesting-Based Nanogenerators

**DOI:** 10.3390/nano13061025

**Published:** 2023-03-13

**Authors:** Shamsu Abubakar, Sin Tee Tan, Josephine Ying Chyi Liew, Zainal Abidin Talib, Ramsundar Sivasubramanian, Chockalingam Aravind Vaithilingam, Sridhar Sripadmanabhan Indira, Won-Chun Oh, Rikson Siburian, Suresh Sagadevan, Suriati Paiman

**Affiliations:** 1 Department of Physics, Universiti Putra Malaysia, Serdang 43400, Selangor, Malaysia; shamsuabubakar05@gmail.com (S.A.); tansintee@upm.edu.my (S.T.T.); josephine@upm.edu.my (J.Y.C.L.); 2 Department of Physics, Yobe State University, Damaturu P.M.B. 1144, Yobe State, Nigeria; 3 Department of Physics, College of Natural Science, Jeonbuk National University, 567 Baekje-daero, Deokjin-gu, Jeonju-si 54896, Jeollabuk-do, Republic of Korea; zainalat@upm.edu.my; 4 Faculty of Innovation and Technology, Taylor’s University Malaysia, No. 1, Jalan Taylor’s, Subang Jaya 47500, Selangor, Malaysia; ramsundar_1994@yahoo.in (R.S.); chockalingamaravind.vaithilingam@taylors.edu.my (C.A.V.); sibisri819@gmail.com (S.S.I.); 5 Department of Advanced Materials Science and Engineering, Hanseo University, Seosan-si 356-706, Chungnam, Republic of Korea; 6 Department of Chemistry, Faculty of Mathematics and Natural Sciences, Universitas Sumatera Utara, Padang Bulan, Medan 20155, Indonesia; rikson@usu.ac.id; 7 Nanotechnology & Catalysis Research Centre, Universiti Malaya, Kuala Lumpur 50603, Malaysia; drsureshnano@gmail.com; 8 Functional Nanotechnology Devices Laboratory (FNDL), Institute of Nanoscience and Nanotechnology, Universiti Putra Malaysia, Serdang 43400, Selangor, Malaysia

**Keywords:** thin film deposition, ZnO nanorods growth, nanogenerator, PFM characterizations, piezoelectric coefficient (d33)

## Abstract

Zinc oxide (ZnO) nanorods have attracted considerable attention in recent years owing to their piezoelectric properties and potential applications in energy harvesting, sensing, and nanogenerators. Piezoelectric energy harvesting-based nanogenerators have emerged as promising new devices capable of converting mechanical energy into electric energy via nanoscale characterizations such as piezoresponse force microscopy (PFM). This technique was used to study the piezoresponse generated when an electric field was applied to the nanorods using a PFM probe. However, this work focuses on intensive studies that have been reported on the synthesis of ZnO nanostructures with controlled morphologies and their subsequent influence on piezoelectric nanogenerators. It is important to note that the diatomic nature of zinc oxide as a potential solid semiconductor and its electromechanical influence are the two main phenomena that drive the mechanism of any piezoelectric device. The results of our findings confirm that the performance of piezoelectric devices can be significantly improved by controlling the morphology and initial growth conditions of ZnO nanorods, particularly in terms of the magnitude of the piezoelectric coefficient factor (d33). Moreover, from this review, a proposed facile synthesis of ZnO nanorods, suitably produced to improve coupling and switchable polarization in piezoelectric devices, has been reported.

## 1. Introduction

The development of scanning probe microscopy (SPM) and atomic force microscopy (AFM) has significantly aided the exploration of nanostructures with diverse piezoelectric coupling properties. Compared to chemical batteries, ZnO-based nanogenerators are environmentally friendly and can provide sustainable electrical energy. The principle of energy-harvesting devices is based on the converse piezoelectric effect, in which the generation of a potential difference is caused by the polarization induced by the electric field. The current generation with an improved piezoelectric response can be increased by improving the growth orientation and density of nanostructures. Controlled synthesis and modification of nanostructured materials have shown great potential in terms of energy and the environment owing to advancements in nanoscience and nanotechnology. Functionality has generally been explored for nanoengineered materials based on their structural, morphological, and chemical composition. In recent times, developments in energy harvesting using micro- and nano-rod-like structures to generate electricity based on direct or converse piezoelectric effects have increased tremendously owing to the simple route of their synthesis, characterization, and fabrication. ZnO is environmentally friendly and meets the requirements of lead-free green technologies. ZnO nanostructures have been reported as metal-based medications that can be used in biomedical applications because of their biocompatibility [1,2]. However, the field of nano-energy harvesting is part of diverse areas of nanotechnology aimed at providing sufficient and sustainable small-scale power solutions, especially with emerging trends in the technology of self-powered devices [3]. The recent literature has focused on optimizing the synthesis parameters related to the fabrication of various ZnO-based piezoelectric generators. Nevertheless, recently, researchers have proposed piezoelectric nanogenerators based on organic polymer materials and their derived piezoelectric copolymers. Inorganic piezoelectric nanogenerator-based devices are inherently poor and rigid in flexibility. Therefore, the energy produced by ZnO nanogenerators (NG) can be enhanced and generated if the arrays are grown on flexible polymer substrates or coated polymer films.

**Figure 1:**
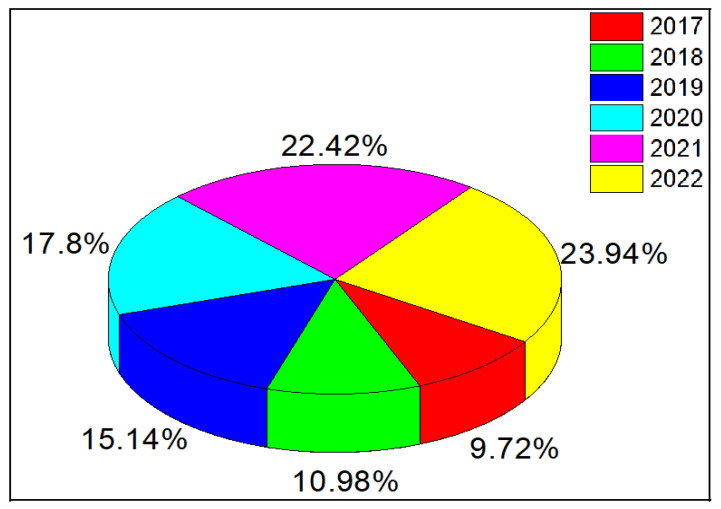
A pictographic chart shows an increasing percentage in the number of publications in the field of piezoelectric ZnO-based nanogenerators.

Recently, piezoelectric nanomaterials that are flexible, affordable, wearable, and inventive have attracted considerable attention for the fabrication of energy harvesters and sensors owing to their high phase content, crystallization, and processing conditions. Electroactive polymers, such as poly (vinylidene fluoride) (PVDF) and its copolymer poly (vinylidene fluoride-co-tetrafluoroethylene) (PVDF-TrFe), have received particular attention among piezoelectric materials. In another study, Zviagin et al. [4] demonstrated that the piezoelectric response of hybrid biodegradable 3D poly(3-hydroxybutyrate) scaffolds coated with hydrothermally deposited ZnO is enhanced. The hybrid biodegradable piezoelectric scaffolds reported in this study are potentially useful in biomedical applications. Consequently, controlling the morphology of ZnO nanorods (NRs) and nanowires during the growth processes has been the focus of our discussion because degrading and limiting the device performance of various nanoelectronics has an intrinsic connection with the initial synthesis and morphological features. Furthermore, it was demonstrated that a piezoelectric nanogenerator fabricated using vertically aligned ZnO nanorods generates a direct current through specific electron dynamics [5,6,7]. The piezoelectric materials used in the fabrication process are relatively topical, with nanoscale confinement provided by localized charge carriers. In recent years, the application of piezoelectric ZnO nanorods and nanowires for energy harvesting has rapidly expanded, resulting in numerous reports in the literature [8,9,10,11,12,13]. The number of publications on ZnO nanogenerators between the year (2017 and 2022) has increased enormously and urgently demands a critical stance to observe the new trend in this direction. According to the source (Dimension Science Database), the percentage of publications in 2022 alone is greater than the total number of publications in previous years. A pictographic chart shows an increasing percentage in the number of publications in the field of piezoelectric ZnO-based nanogenerators, as shown in Figure 1.

Several studies have reported the synthesis and growth of various ZnO nanostructures. Semenova et al. [7] successfully demonstrated how to grow a ZnO nanostructure on a zinc oxide seed layer using a spin-coating technique and hydrothermal synthesis, which describes epitaxial growth configurations via solution growth. They described it as a low-cost, transparent, and simple method. The main idea is to establish a foundation for fabricating ZnO as an active component of a piezoelectric nanogenerator with different capabilities for future applications. It also demonstrates the growth of vertically oriented ZnO nanorods on the sputtered seed layer by radio frequency (RF) magnetron methods because of its efficiency in obtaining film seeds with a small crystallite grain size. The synthesis of orderly-grown nanostructures with high-density arrays has prompted the need for their dynamic integration into nanoelectromechanical systems to improve their actuation performance for various applications. However, many materials have different surface energies owing to the differences in the surface texture associated with the features of the grains in their respective domains [14]. In other words, the substrates used to deposit nanostructures had a more significant influence on the subsequent morphological characteristics of the nanorods and nanowires [15]. Ding J. et al. [16] investigated the impact of various substrates on the geometric morphology of ZnO nanorod arrays fabricated by the hydrothermal method. An ultrathin ZnO seed layer was deposited by atomic layer deposition, and the substrates included silicon, glass, indium tin oxide (ITO), and boron-ZnO films. The scanning measurements confirmed that the orientation and alignment of the nanowires were controlled by the surface texture of the substrate and the roughness of the deposited film seed layers, respectively. According to some studies [17,18,19], the thickness and roughness of the seed layers have a greater impact on the alignment of the nanorods than the texture of the substrate. AFM micrographs have been developed in the field of nanotechnology for various applications [20,21,22,23,24]. Energy harvesting or energy scavenging is defined as a process that takes energy from the surrounding energy sources, accumulates the energy, and then stores it for later use. Energy harvesting technology enables the generation of electrical energy from waste energy sources that are always and everywhere present, such as heat, liquid, and vibration. It is the most effective way to respond to energy shortages and generate sustainable energy sources from the environment compared to traditional batteries. With the global energy crisis and environmental concerns, many types of research and technology have already focused on energy harvesting technologies, such as solar, wind, geothermal, and hydroelectric power. Energy harvesting can be categorized into macro- and micro-energy harvesting technologies. Therefore, many energy-harvesting technologies, such as self-power sources and nanogenerators, have been studied. This review focuses on the growth conditions for the synthesis of improved ZnO nanostructures with controlled morphology, dimensional orientation, and high crystal assembly density. Different synthesis methods such as chemical vapor deposition (CVD), thermal evaporation, RF sputtering, metal-organic chemical vapor deposition (MOCVD), electrodeposition, and chemical bath deposition have been used for the synthesis of nanostructured ZnO. Specifically, chemical bath deposition (CBD) is considered one of the simple and economical techniques for the synthesis of ZnO nanostructures on different substrates using low temperatures. The CBD method has the potential to produce diverse ZnO nanostructures, including nanorods (NRs), nanowires, nanotubes, and nanobelts [25]. Zinc oxide nanorods, nanowires, and nanotubes (ZnO-NRs, NWs, and NTs, respectively) have attracted considerable interest over the past few years owing to their unique physicochemical and piezoelectric electromechanical properties. Notably, ZnO nanorods are among the 1D nanostructures that are currently being used in piezoelectric devices, such as nanogenerators and other nanoelectromechanical systems.

## 2. Structural, Electronic, and Piezoelectromechanical Properties of ZnO

**Figure 2:**
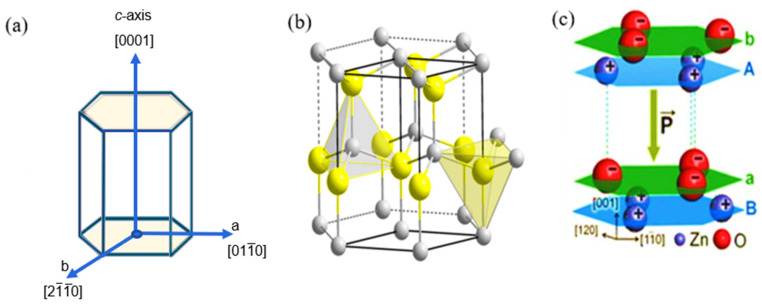
(**a**) Crystal structure of hexagonal wurtzite ZnO along the polar axis with different crystallographic facets. (**b**) The hexagonal wurtzite structure of ZnO with the tetrahedron coordination; O (grey spheres) and Zn (yellow spheres). (**c**) Alternate layers of positive and negative ions in ZnO with a non-centrosymmetric structure, which causes spontaneous polarization. Reprinted from Refs. [34,35,36].

ZnO is a well-known piezoelectric material that has been studied because of its importance, suitable atomic crystal configurations, and potential applications in piezoelectric and optoelectronic devices [26,27]. However, it is a semiconductor with a wide bandgap (~3.37 eV), high excitation binding energy (~60 meV), and the ability to generate the desired electron mobility [28,29]. Haffad S. et al. [30] studied the structural and electronic properties of ZnO nanowires with different geometrical shapes and sizes by density functional theory calculations. In particular, the ground-state properties predicted in their work showed that the atomic energies and relaxations linked to the electronic properties of ZnO nanostructures depend largely on their shape and orientation. It was found that hexagonal nanorods underwent surface relaxation with slight changes in the lattice configurations along the nanorod’s perpendicular axes and side directions. In hexagonal ZnO, each lattice cell comprises structures that are tetrahedrally centered with oxygen or zinc atoms. Fundamentally, the ground state orbitals of the oxygen and zinc atoms in the tetrahedral structure of ZnO are responsible for hybridization in the structure. The electronic arrangement of outermost orbits for oxygen is composed of 1s^2^ 2s^2^ 2p^4^, and 2s and 2p orbitals crossbreed to form sp^3^, which has four orbitals that contain eight electrons. The sp^3^ has a tetrahedral structure with six filled electrons; therefore, two electrons are required to be stable. The electronic orbits of the zinc atoms are 1s^2^ 2s^2^ 2p^6^ 3s^2^ 3p^6^ 4s^2^ 3d^10^. The 4s and 4p orbitals hybridize to shape the sp^3^ orbitals, which have been occupied by two electrons. To form a stable zinc structure, six electrons need to be shared by oxygen, and oxygen receives two electrons shared by zinc. In this hybrid, a hexagonal ZnO structure was obtained [30]. This finding proved the dependence of the mechanical properties on the ZnO nanorods. Recent advances in piezoelectric nanogenerators and actuators have prompted the need to adequately integrate new techniques to improve the synthesis conditions of ZnO nanostructures with various morphological architectures and crystal orientations suitable for energy harvesting. Among the known materials, quasi-dimensionally oriented nanostructures, such as nanowires, nanorods, nanotubes, nanobelts, and nanoflowers, have been synthesized based on controlled growth deposition [31,32,33]. At present, ZnO has attracted the interest of researchers owing to its utilization through a facile chemical approach. Schematic representations of the ZnO crystal structure along the polar axis with different unit cells of Zn^2+^ and O^2−^ ions between the stacked layers are shown in Figure 2.

Typically, a binary semiconducting material exhibits piezoelectric properties in a crystallized hexagonal wurtzite structure [34,35,36]. The physical dimension of unit cells in a crystal lattice of ZnO (lattice constants) are 𝑎 = 0.3296 nm and 𝑐 = 0.526065 nm. Normally, ZnO crystals grow into wurtzite hexagonal or zinc-blend structures with O^2−^ and Zn^2+^ ions forming alternate planes, as shown in Figure 2. The crystal lacks symmetry around its central axis because of this ion arrangement, and the material exhibits piezoelectric properties. Furthermore, the net positive and negative charges on the basal planes ( 0001 ) and ( $000\bar{1}$ ) are caused by the positive charge on zinc ions and the negative charge on oxygen ions, respectively. With its polarization vector along the c-axis, the crystal becomes polar [35,36,37]. A surface with a net electric charge is inherently unstable. However, the basal faces of the ZnO crystals were stable and did not have any surfaces; therefore, they remained flat. The reasons for the stability of the polar surfaces of the ZnO crystals are unclear. However, extensive studies have been conducted in this area [38,39,40]. Apart from the polar basal planes, the facets ( $2\bar{1}\bar{1}0$ ) and ( $01\bar{1}0$ ) in the wurtzite hexagonal ZnO crystal are stable and have low surface energies because they are non-polar [41,42,43]. During the synthesis of ZnO, the anisotropic growth of the crystal, in which the ions in the precursor are attached preferentially to the individual polar face of the crystal, results in the formation of different orientations. This preferential growth is determined by the amount of surface energy and the number of active sites per unit area of the face. As a result, crystal growth occurred along the direction normal to the high-energy face, whereas the lower-energy faces expanded. In general, ZnO crystals grow faster in the ±[0001] and ±[0110] orientations of polar faces. This is probably an unavoidable reason for both the vertical and slanted orientations during growth. The majority of research has focused on zinc oxide because nanostructures can be synthesized using low-temperature techniques, and their crystallographic orientations can be aligned without poling.

## 3. ZnO Seed Layer Deposition and Thickness Dependence

Thin-film deposition or seeding has been widely used [44,45] to support the growth of various nanostructures on a substrate by initiating adhesive contact between the surface of the substrate and the deposited atoms. Normally, ZnO nanostructures are synthesized either in the solution or gaseous phase. A sol-gel method with hydrated zinc acetate solutions is used in solution phase synthesis together with NaOH or tetramethylammonium hydroxide (HMTA) [46] and spray pyrolysis [47,48,49,50]. Physical vapor deposition (PVD), chemical vapor deposition (CVD), metal-organic chemical vapor deposition (MOCVD), and vapor-liquid-solid (VLS) are examples of gaseous-phase synthesis techniques [51,52,53,54,55,56,57]. ZnO can be deposited on different substrates using various chemical and physical deposition techniques, such as sputtering [58,59,60]. The oxygen anions (O^2−^) and zinc cations (Zn^2+^) nucleate over the seed layers, forming additional planes of stacked deposits of oxygen and zinc and subsequently forming aligned hexagonal rods along the c-axis. Many studies have been conducted to investigate the different properties of ZnO nanostructures [61]. Using a low-temperature solution technique, Wu et al. [62] synthesized vertically aligned ZnO nanowire arrays on ZnO/glass substrates. Radio-frequency sputtering was used to develop seed layers of different thicknesses on the glass substrates. The thickness of the ZnO seed layers varied from 20 nm to 1000 nm. According to their findings, the sizable impact on the shape of ZnO nanowires revealed an increase in diameter from 50 to 130 nm, whereas the density decreased from 110 to 60 µm^−2^ when the seed layer thickness was controlled. Supawadee et al. [63] used the hydrothermal approach to grow zinc oxide nanorods (ZnO-NRs) on substrates with ZnO seed layers at 90 °C for 6 h. ZnO seed layers were deposited with various thicknesses, and their impact on the nanorods was comprehensively examined. Field-emission scanning electron microscopy and X-ray diffraction were used to study the morphology and crystal structure of the ZnO samples. The results revealed that the thickness and surface roughness of the film seed layers directly affected the crystallinity of the ZnO NRs. Table 1 shows the growth deposition parameters of sputtered ZnO thin films on substrates with varying surface roughness values and thicknesses.

Son et al. [68] investigated the effect of ZnO thin films on vertically aligned ZnO nanorod chemical bath deposition. The seed layer thickness was varied in the experiment. ZnO nanorods were grown on a thin film at 90 °C using CBD. The surface granularity and vertical roughness of the ZnO thin films aided the vertical growth of ZnO nanorods. The average grain size and surface roughness of the ZnO film increased as the film thickness increased, increasing both the average diameter and average length of the vertically produced ZnO nanorods. The average diameter of ZnO nanorods was found to be extremely close to the average grain size of ZnO thin film, confirming ZnO film’s role as a seed layer for ZnO nanorod vertical growth. Data on the development conditions of sputtered ZnO seed layers deposited on substrates with varying surface roughness and thickness values are summarized in Table 1. In most previous reports, the ZnO seed layer was typically deposited onto the substrate before the growth of the vertically aligned ZnO nanorods.

**Figure 3:**
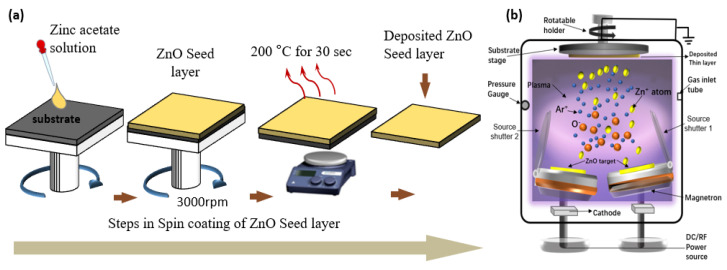
(**a**) Schematic illustration of the ZnO seed layer deposition by a spin coating method and (**b**) magnetron sputtering deposition.

Many experimental studies have demonstrated a strong interest in thin-film deposition to achieve high-quality film seeds using various PVD techniques [69]. For instance, RF-magnetron sputtering deposition [70] has been known for decades owing to the high deposition rate, purity, and homogeneity of crystallite grains. Dang et al. [71] used rf-magnetron sputtering at room temperature (RT) with an installed metallic zinc target in an argon-oxygen gas mixture to deposit ZnO thin films. The reactive gases, power, and substrate temperature were varied. To optimize these deposition parameters, an optimized approach was used to consider their interdependence. Their discoveries revealed that plasma power and gas pressure have the greatest influence on film crystallization and stress and that post-deposition annealing improves film quality. The plasma power, O_2_/Ar gas ratio, and gas pressure were among the interdependent deposition parameters that were investigated. The RF power was adjusted between 225 and 275 W, the O_2_ concentration varied between 5% and 25%, and the pressure varied between ~0.7 and 1.3 Pa. Low-cost fabrication of films requires a higher deposition rate. The maximum deposition rate achieved was 22.4 nm/min, allowing for the deposition of thick films over a short period. The effect of the deposition parameters on the ZnO crystallite size has also been studied [72,73]. Pranav et. Al. [74] reported that the magnetron sputtering technique was used to deposit nano-structured zinc oxide thin films on a corning glass substrate. Different argon/oxygen gas ratios were used to deposit ZnO films. X-ray diffraction (XRD) was used to characterize the ZnO thin films and clearly showed the existence of the (100) and (002) peaks. A higher RF power results in larger crystallite sizes, which promote grain formation by increasing the energy of the atoms arriving at the surface. Increases in the deposition pressure were also associated with larger crystallite sizes since higher pressure correlates to a higher number of atoms arriving at the surface, improving the growth efficiency [74]. Numerous studies have been published on the deposition of ZnO seed layers on substrates and their effects on the quality of nanorods [75,76]. Gas-phase deposition and liquid-phase synthesis techniques are the two main classes of these techniques [77]. In terms of methods, cost, environmental consequences, and efficiency, each technique has advantages and disadvantages. In general, gas-phase deposition processes require stringent conditions, such as high temperatures or vacuum, which require the use of advanced technology and expensive equipment. These approaches provide significant control over the deposition parameters, which can yield the desired result [77,78,79]. However, liquid-phase deposition techniques are simple and inexpensive and provide limited control over the synthesis parameters that affect the quality of the grown nanostructures [80]. Figure 3 shows a schematic illustration of ZnO seed layer deposition using a spin-coating method and magnetron sputtering.

According to some studies, increasing the amount of O_2_ used during deposition resulted in smaller crystallite sizes that were much smaller than the thicknesses of the formed films. Because there was more O_2_, the Ar and O_2_ atoms collided more frequently, resulting in less growth. AFM was used to examine the surface morphology and roughness of the films over a cross-sectional area of 1 µm^2^. The root-mean-square (RMS) surface roughness values of these films were less than one nanometer, indicating that they were smooth. It was also discovered that annealing had little to no effect on surface roughness. On the other hand, ZnO seed particles on the substrate can be annealed at a specific temperature to increase their adhesion to the substrate, thereby enhancing the growth and alignment of the nanorods [68,81,82]. Baruah et al. [83] investigated the effect of seeding glass substrates with zinc oxide nano-crystallites on the hydrothermal growth of ZnO nanorods from a zinc nitrate hexahydrate and hexamethylenetetramine solution at 95 °C. Seeding was performed with pre-synthesized ZnO nanoparticles in isopropanol with diameters of 6–7 nm, as well as the direct synthesis of ZnO nanocrystallites on the substrates via hydrolysis of a pre-deposited zinc acetate layer. The nanorods grown on the ZnO nanoparticle seeds had uniform diameters across the substrate, although they were not vertically aligned uniformly. Thin films are widely used in various device applications owing to their electrical, mechanical, optical, and thermal capabilities [83]. The RF sputtering deposition of fabricated ZnO seed layers on the various substrates aided the growth mechanism of the aligned nanorods and nanowires. For the hydrothermal synthesis of ZnO nanorods, Liu et al. [84] employed a ZnO thin film prepared using the Pulsed Laser Ablation (PLA) approach as a seeding layer. In comparison, Li et al. [51] demonstrated that a low-temperature solution technique was used to grow vertically aligned crystalline ZnO nanowire arrays on seeded ZnO/glass substrates. Radio-frequency sputtering was used to create ZnO seed layers of various thicknesses on the glass substrates. During the growth, the chamber’s working pressure was 3.0 × 10^2^ Torr, the RF power was 200 W, and the O_2_/Ar gas flow ratio was maintained at ~1/10. During deposition, no substrate heating was used. The seed layer thicknesses were adjusted from (20, 240, 500, and 1000 nm).

Improved morphological control of the ZnO nanowire arrays results in increased carrier mobility in ZnO-based hybrid systems. The thickness of the seed layers significantly influences the growth of the ZnO NRs [85]. Furthermore, as the thickness of the ZnO seed layer increased, the NW diameter of the NW, while the density of the NW decreased. The surface roughness increased as the thickness of the ZnO sheet increased.

## 4. Characterization of ZnO Nanostructures Synthesized via an Aqueous Chemical Route

**Figure 4:**
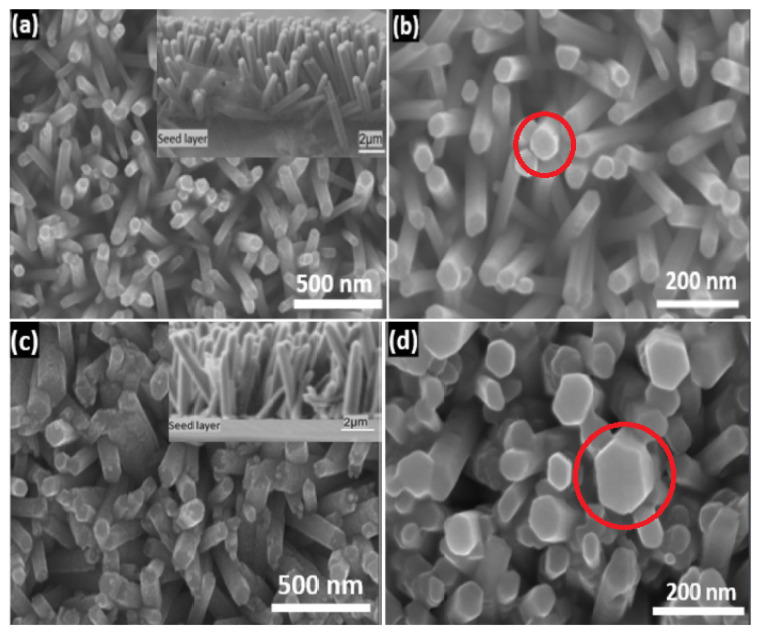
(**a**–**d**) High and low magnification FE-SEM images of ZnO NRs growth on silicon and glass substrates. The insets at the upper right-hand corner in (**a**–**c**) show the cross-sections. Sketch red circles in (**b**,**d**) show a hexagonal polar face in [0001] direction. Reprinted with permission from ref. [89]. Copyright 2021 Elsevier.

Several previous studies have reported the synthesis and growth of ZnO nanostructures with multi-dimensional properties [85,86,87]. If the exotic features of these materials are continually investigated, they will play a far larger role in many electronic components. Fundamentally, the crystal structure of ZnO tends to maximize the area of the ( $2\bar{1}\bar{1}0$ ) and (01 $\bar{1}$ 0) facets, growing in the [0001] direction. This means that the last two facets are nonpolar surfaces with lower energies than polar surfaces. As a result, one-dimensional ZnO nanostructures are widely diverse in shape [87,88]. Figure 4 shows the FESEM images of ZnO nanorods on Si and glass substrates with hexagonal surfaces.

**Figure 5:**
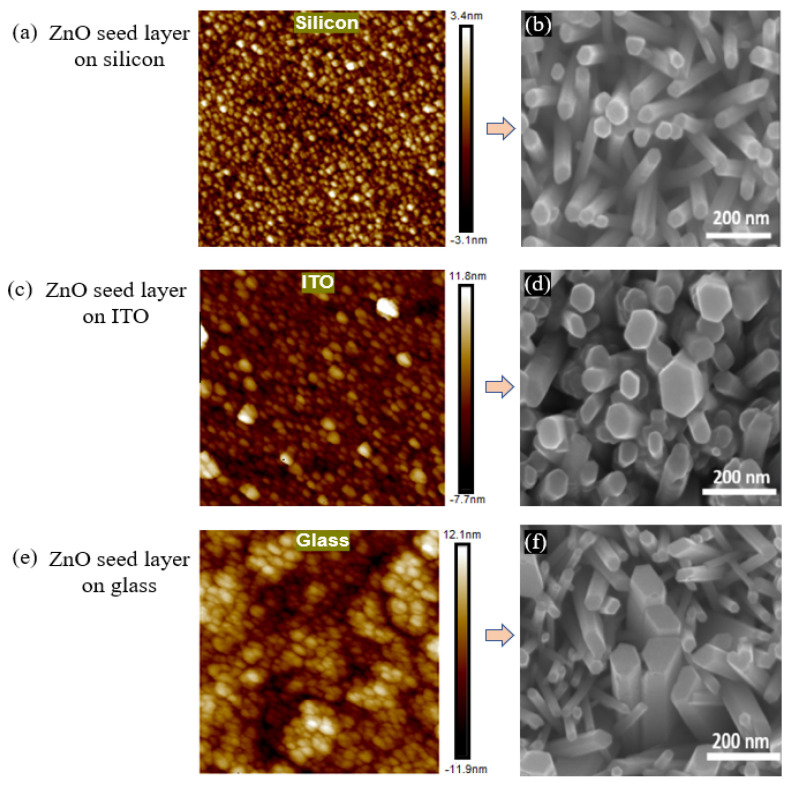
AFM images of the seed layer and FESEM images of ZnO NRs grown on different substrate materials (**a**,**b**) silicon, (**c**,**d**) ITO, and (**e**,**f**) microscopy glass slides. Reprinted with permission from ref. [89]. Copyright 2021 Elsevier.

In this work [89], the results observed similar grain size orientations of the seed layer on three different substrates, as shown in Figure 5 (a,b) silicon (c,d) ITO, and (e,f) glass slides. As can be observed from Figure 5b,d,f, the morphology of nanorods grown on different substrates seemed to have different crystal orientations. This shows that the seeded substrate layer had little influence on the morphology. However, for the subsequent growth of ZnONRs, pre-synthesis chemicals of analytical-grade standards are used, mostly without further purification. Therefore, cleaned substrates are required before film seeding or coating.

**Figure 6:**
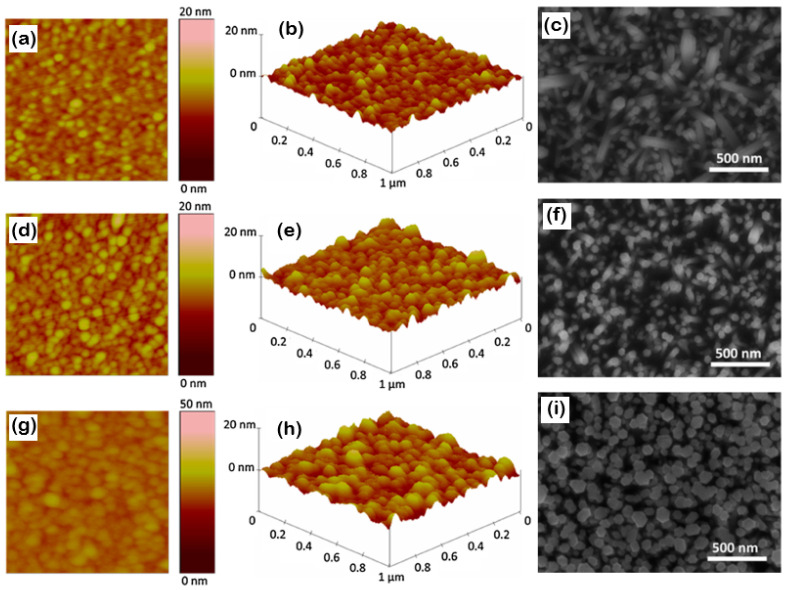
AFM (2D, 3D images) for ZnO seed layer sputtered for 10 min, 15 min, and 20 min, (**a**–**h**) and (**c**) top view FE-SEM view of ZnO nanorod arrays grown for (**c**) 10 min, (**f**) 15 min, (**i**) 20 min, respectively. Reprinted with permission from ref. [68]. Copyright 2016 Elsevier.

However, under certain conditions, the (0001) polar face of the hexagonal wurtzite structure of ZnO with c-axis crystal orientation shares orbital features with the hexagonal rings of carbon-based materials such as graphene. Huy Q. et al. [90] have proven that the ZnO structure has graphene-like characteristics. Graphene–zinc oxide (g-ZnO) composites also have good prospects as valuable materials for many applications. It is worth noting that the polar surfaces of ZnO are relatively stable and undergo little surface reconstruction under strain. When ZnO and graphene nanocomposites are joined, the problem of lattice mismatch can be significantly reduced. Several methods have been described for producing well-aligned ZnO nanorods from aqueous solutions. Conventionally, an aqueous solution is prepared for the growth of ZnO nanorods by dissolving a certain molar ratio of zinc nitrate hexahydrate $\left(\mathrm{Zn}\left({\mathrm{NO}}_{3}\right)\;6H_{2}O\right)$ together with hexamethylene tetraamine (HMTA) [91,92,93]. This synthetic pathway and its modifications have been widely used to improve the orientation, crystallinity, diameter, and aspect ratio of nanorods. The chemical pathway leading to the formation of ZnO from hexamine and zinc nitrate hexahydrate involves the dissolution of hexamine into formaldehyde and ammonia during prolonged heat treatment (5–6 h at 90 °C), followed by the formation of hydroxyl ions from ammonia [94]. Ammonia and hydroxyl ions react with Zn^2+^ ions liberated by dissolving $\left(\mathrm{Zn}\left({\mathrm{NO}}_{3}\right)\;6H_{2}O\right)\;$ to form $\;\mathrm{Zn}\left({\mathrm{NH}}_{3}\right)\begin{array}{c} 2+\\ 4 \end{array}$ complexes, which serve as precursors for ZnO nanorods. It is predicted that if this pathway is shortened, the reaction time, temperature, and precursor concentration are reduced. To shorten the reaction time, the degree of supersaturation should be reduced, and homogeneous nucleation suppressed. Table 1 summarizes the deposition-growth parameters for the ZnO seed layer and ZnO nanorod arrays synthesized via chemical bath deposition and hydrothermal techniques [95]. The AFM images of the ZnO seed layers deposited by RF sputtering maintained a steady growth rate of 8 nm/min, and the sputtering time was varied to obtain ZnO seed layers of various thicknesses. According to Son et al. [68], the seed layer grain sizes were composed of small grains, as shown in the AFM images above, and this was dependent on the sputtering time. Small ZnO grains with characteristic root-mean-square (RMS) roughness were observed on the surface of the 10 min sputtered ZnO thin film, with average grain sizes of ~0.912 nm and 70 nm, respectively. Both the RMS roughness and average grain size increased when the deposition period was extended to 15- and 20-min. Figure 6 depicts the AFM images (2D and 3D images) of the ZnO seed layer.

**Figure 7:**
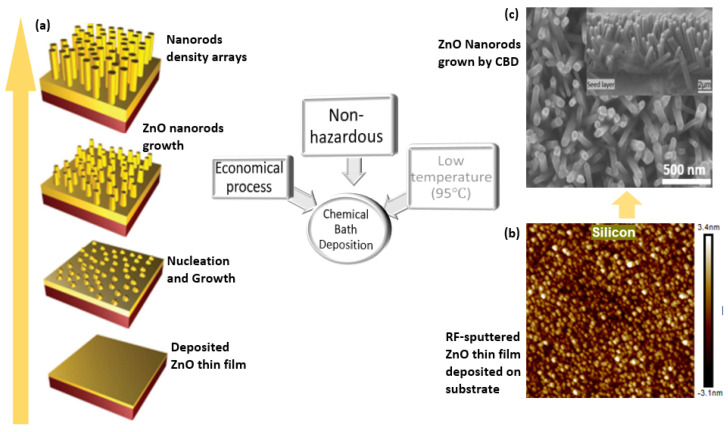
Schematic illustration of (**a**) ZnO seed layer deposition, followed by ZnO nanorod arrays synthesized by chemical bath method assisted (**b**) sputtered ZnO seed layer deposited on the substrate (**c**) FESEM image of ZnO nanorods grown using CBD. Reprinted with permission from ref. [89]. Copyright 2021 Elsevier. Reprinted from ref. [102].

AFM line-scan profiles can confirm the increase in surface roughness and average grain size, as reported in the literature [16,68,76,96,97,98,99,100,101,102]. The rough grain structure of the ZnO seed layer contributed to the vertical growth of the ZnO nanorods during subsequent CBD growth. Figure 7 also shows a schematic illustration of ZnO seed layer deposition followed by ZnO nanorod arrays synthesized using a chemical bath method. There were significant findings on the crystal morphology evolution, where a certain range of temperatures, zinc nitrate, HTMA precursor concentration effect, and seed layer thickness were reported to improve the crystallinity and growth alignment of NRs and NWs [103,104]. These results provide a further understanding of ZnO nanostructures for future applications in energy harvesting and other related fields.

## 5. Growth Characteristics of Nanorods and Nanowires

One-dimensional (1D) nanostructures (rods and wires) have been applied in optoelectronic devices because of their fundamental importance in a variety of advanced technologies [105], gas sensors [106], solar cells [107,108], and photocatalysis [109,110]. Highly oriented arrays of one-dimensional (1D) ZnO nanostructures are of great importance for the development and enhancement of the output performance of piezoelectric nanogenerators [10,26,111,112,113,114]. Several technologically advanced approaches have been developed for the fabrication of well-aligned 1D ZnO nanostructures [115,116]. The simplest and most direct route to prepare 1D nanostructure arrays is the chemical bath deposition or hydrothermal growth techniques [117,118,119]. The seed-induced hydrothermal method was developed by Vayssieres et al. [120] to produce ZnO nanorod arrays on silicon and glass substrates in high-pressure autoclaves. The electrochemical deposition has recently been used to grow ZnO nanorod arrays on transparent conductive substrates [121]. However, certain controlled synthesis methods effectively increase the orientation, shape, and crystallinity of ZnO nanostructures [48,122,123,124]. Although seedless growth in complex solutions complicates the fabrication process, this unique chemical technique slightly improves the density of ZnO nanowire (NW) arrays by controlled growth [125]. The density of ZnO NWs can be controlled by adjusting the precursor concentration and the impact of the growth temperature and time. In contrast to previous claims [74], this novel synthesis method has shown that ZnO NW arrays can grow on clean and flat surfaces, including polymers, glasses, semiconductors, and metals. This technology has been described as a new, low-cost, time-efficient, and scalable method for producing ZnO NW arrays for use in field emission, vertical field-effect transistor arrays, nanogenerators, and nanopiezotronics. Subsequently, Kyung et al. [126] reported the growth of ZnO nanorods on ZnO seed layers synthesized by sol-gel solution with varying annealing temperatures on diverse substrates (glass and silicon fluorine-doped tin oxide (FTO)-coated glass). Their key finding demonstrates that NRs alignment and morphology can be improved in a specific temperature range. In total, 0.15 M zinc acetate dihydrate (precursor) was dissolved in ethanol and stirred for 1 h at 60 °C and room temperature (RT) to prepare the seeded layer solutions. Subsequently, after spin-coating, the substrate was annealed at different temperatures (150–450 °C) for 30 min. The thickness of the ZnO layer was fixed to 20 nm. For the growth of nanorod, 0.01 M zinc nitrate hexahydrate and 0.01 M HMT were prepared using the hydrothermal method. AFM and field emission scanning electron microscopy (FESEM) were used to examine the morphologies. The results showed that all ZnO nanorods had hexagonal wurtzite structures and were randomly oriented.

**Figure 8:**
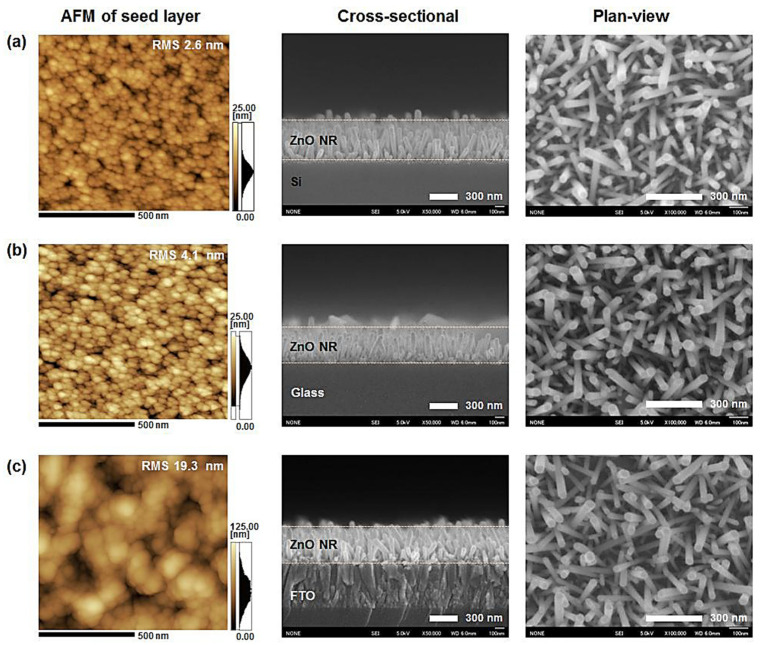
AFM images of the seed layer annealed at 350 °C and FESEM images of ZnO NRs grown for 3 h on different substrate materials: (**a**) silicon, (**b**) glass, and (**c**) FTO/glass. Reprinted from ref. [126].

The AFM micrographs also show that the particle size increases with increasing temperature, and FESEM also shows that the length of the NRs increases when the annealing temperature increases from 150 °C to 350 °C, but then decreases at 450 °C, as shown in Figure 8. This shows that the growth rate of ZnO NRs on the seed layer increased with increasing annealing temperature within a specific range, which affected the crystallinity of the seed layer and improved the growth of NRs [126]. The fascinating trend in material technology is to control the nanostructure synthesis parameters to form arrays on seeded substrates in order to obtain an efficient, high-performance device. Low-temperature chemical bath deposition and hydrothermal synthesis have facilitated a simple route for the growth of many nanostructures with minimal environmental impacts. Therefore, synthesis, fabrication, and characterization of these nanomaterials have become commonly known techniques.

Up till now, mostly reported chemical compositions for the growth of ZnO NWs and NRs by CBD or hydrothermal technique remained a distinctive approach of coalescing zinc nitrate $\left(\mathrm{Zn}\;({\mathrm{NO}}_{3}{)}_{2}\right)\;$ with hexamethylenetetramine (HMTA) in deionized water and heating them over a certain temperature range from 60 °C to 95 °C. In an adjustable milli-molar range of chemical precursor concentration, the growth of ZnO NWs and NRs will have occurred under the fundamental chemical reaction Equations (1)–(8) as follows: The growth mechanism involves the thermal decomposition of hexamine, which produces hydroxyl ions, which react with ${\mathrm{Zn}}^{2+}$ ions to form ZnO as an end product [68].

(CH_2_)_6_N_4_ + 6H_2_O ↔ 6CH_2_O + 4NH_3_ (1)

(CH_2_)_6_N_4_ + Zn^2+^ ↔ (Zn (CH_2_)_6_ N_4_)^2+^ (2)

NH_3_ + H_2_O ↔ NH_4_^+^ + OH^−^ (3)

Zn^2+^ + 4NH_3_ ↔ Zn (NH_3_)_4_^2+^ (4)

Zn^2+^ + 4OH^−^ ↔ Zn (OH)_4_^2−^ (5)

Zn (NH_3_)_4_^2+^ + 2OH^−^ ↔ ZnO + 4NH_3_ + H_2_O (6)

Zn (OH)_4_^2−^ ↔ ZnO + H_2_O + 2OH^−^ (7)

(Zn (CH_2_)_6_ N_4_)^2+^ + 2OH^−^ ↔ ZnO + H_2_O + (CH_2_)_6_N_4_ (8)

Several properties have been discovered to have a significant influence on the morphology, aspect ratio, and overall efficiency of as-synthesized ZnO nanorods. However, the optimization of these features is dependent on the intended applications of these nanomaterials. However, a brief discussion of the effects of these parameters on morphology and efficiency is provided. Some of the important parameters involved in the synthesis are the effects of precursor concentration, growth time and temperature, hexamine, and post-deposition annealing. Table 2 displays the experimental data and deposition-growth parameters for the ZnO seed layers and ZnO nanorod arrays created using chemical bath deposition and hydrothermal techniques, respectively.

### 5.1. Effect of Precursor Concentration

**Figure 9:**
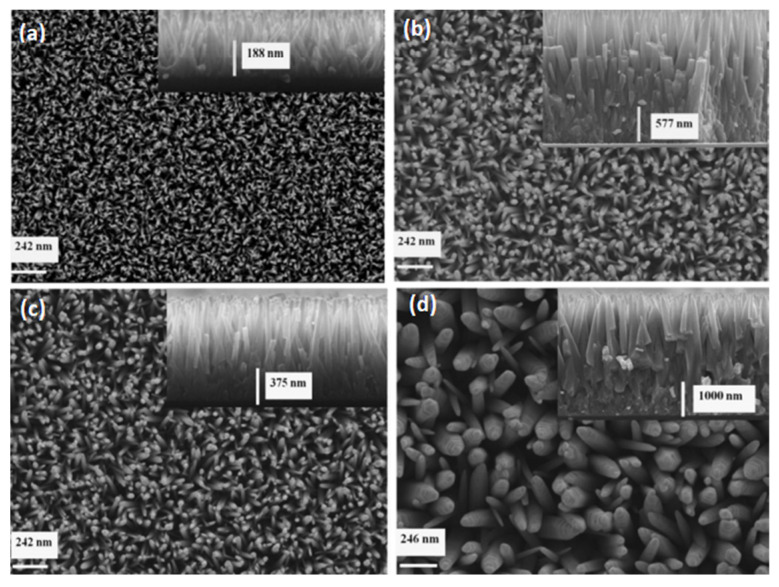
(**a**–**d**) FESEM images of ZNRs grown with different concentrations of Zn(NO_3_)_2_·6H_2_O and NH_4_OH, respectively. The insets are the corresponding cross-sectional images of the ZnO NRs. Reprinted with permission from ref. [103]. Copyright 2012 Elsevier.

The precursor solution used in hydrothermal growth or chemical bath deposition provides ${\mathrm{Zn}}^{2+}$ ions for the formation of ZnO nanostructures with varying crystal dimensions. The growth rate is affected by the precursor concentration as well as other parametric variables [131]. G. Amin et al. [132] investigated the effects of precursor concentration, pH, growth time, and temperature on the morphology of ZnO nanostructures grown using the hydrothermal method. They discovered that the initial pH fluctuated throughout the growth process, eventually settling at neutral pH. Furthermore, the precursor concentration, growth time, and temperature affected the morphology and size of the ZnO nanostructures, which ranged from nanowires to nanorods and even a film-like structure. However, because these studies were repeatable, it is believed that the morphology and structural properties of the grown materials can be controlled by simply changing the growth conditions mentioned above to achieve the desired nanostructures for specific applications in piezoelectric nanogenerators (PENG). As previously investigated by [51,52], the growth characteristics of hydrothermally synthesized 1D zinc oxide nanocrystals are related to the precursor solution concentration, growth period, hexamine functions, synthesis temperature, precursor pH, and seeding layer deposited on the substrate. They discovered that the size, shape, orientation, and growth rate were all highly influenced by several synthesis and growth parameters using a simple and low-temperature hydrothermal approach. Some of these effects have been mentioned in the literature more than once without further explanation of their implications for applications in piezo-electromechanical systems. Urgessa et al. [103] also studied how precursor concentration affects the formation of zinc oxide nanorod arrays on pre-treated substrates, as shown in Figure 9. They successfully synthesized ZnO NRs using the CBD technique on Si substrates that had been pre-treated with a ZnO seed layer by spin coating. This study demonstrated that systematically changing the precursor concentration had a significant impact on the diameter and aspect ratio of the grown ZnO NRs. XRD examination revealed that the low-cost CBD provided good crystallinity.

Controllable growth of the desired functionality is a vital part of the ZnO nanorod technology. A fundamental understanding of the effects of the preparation variables and growth mechanisms is required to optimize the growth of ZnO NRs and NWs for the desired functionality. While studying the effect of the precursor concentration on the shape of ZnO nanorods synthesized on a substrate, Gou et al. [128] also previously discovered that the diameter of the hexagonal nanorod is significantly affected by the precursor concentration. Growth parameters, such as substrate pre-treatment, growth temperature, deposition duration, and precursor concentration, were also shown to have a significant impact on the morphology and alignment ordering of ZnO nanorod arrays. The average rod diameter decreased with decreasing concentration. The precursor concentration reduces the average rod diameter by one-third in a high-concentration regime; however, in a low-concentration regime, a large change in concentration results in only a minor change in rod thickness. Another exciting discovery is that the diameter range of the rods in the low-concentration regime was significantly narrower than that in the high-concentration regime. Pourshaban et al. [133] examined the effect of seed layer sol-gel concentration on ZnO nanorod arrays synthesized by chemical bath deposition in a variety of ways. Among all the parameters influencing the chemical bath deposition method, this study highlighted the importance of seed layer quality in controlling the structural, morphological, and other dimensional features of ZnO nanorods. This relationship has been linked to the formation of many nucleation sites in high-concentration precursor solutions. These observations indicate a lack of consistency, which is understandable given the complex interrelationships among the many parameters involved in the synthesis process. However, in other studies, the growth rate of the ZnO nanorods increased both laterally and longitudinally as the precursor concentration increased. Based on the high surface area efficiency of the as-synthesized nanorods, Mahmood et al. [52] discovered that nanorods synthesized in a zinc-containing aqueous solution were relatively more effective. This is in contrast with the findings in [129], which showed that uniformly aligned ZnO nanorods can be obtained at a starting concentration of 0.025 M.

### 5.2. Growth Time and Temperature

In most experimental reports, the morphological characteristics of ZnO nanostructures exhibit a similar trend in the variation of synthesis parameters, especially growth time and temperature, which have been observed due to thermochemical decomposition [134]. Polsongkram et al. [124] made observations about how the initial precursor concentration and reaction temperature can be used to achieve selective growth of nanorods ranging from a thin to a wider diameter. When the synthesis process was carried out at a lower temperature (60 °C), thick ZnO nanorods with thick shapes were obtained by varying the temperature. The growth rate is faster in the [2110] direction than in the [0001] direction. These findings show that the composition and temperature of the ZnO nano/microrods play a crucial role in their fabrication. In addition, these findings may lead to the development of new nanodevices through an understanding of the growth behavior of ZnO nanorods. Well-aligned ZnO nanorod arrays synthesized using a low-temperature wet-chemical bath deposition (CBD) technique under various conditions have been analyzed in a previous study [51,103]. Another study [129] investigated the size and aspect ratio of synthesized ZnO nanorods versus temperature using cross-sectional and corresponding SEM images of ZnO nanorods grown at *T* = 80 °C, 85 °C, and 90 °C. According to these findings, dense arrays of ZnO nanorods with hexagonal wurtzite structures were vertically aligned and uniformly dispersed. The basic idea behind synthesizing densely oriented nanorods by varying the precursor growth temperature, concentration, and time on the nanorods is to achieve a better morphology, orientation, and aspect ratio for appropriate applications.

### 5.3. Role of Capping Agents (HMTA)

The role of hexamine in the formation of ZnO nanorods remains unknown. The most well-known function of hexamine is to provide OH ions for ZnO formation. On the other hand, Romain et al. [135] use CBD to study the effects of HMTA on the nucleation and radial growth of ZnO nanowires. The influence of the chemical precursors in solution on the structural characteristics of ZnO NWs produced by CBD was thoroughly investigated using a seed layer with a specific structural topology. The structural properties of the ZnO NWs/NRs produced by changing the non-equimolar ratios of Zn (NO_3_)_2_ and HMTA over a wide range and substituting HMTA with NH_3_ demonstrate HMTAs many roles of HMTA. The effects of HMTA on the length of ZnO NWs were observed to improve as the ratio of (Zn(NO_3_)_2_): (HMTA) was reduced from 4 to 0.66 (i.e., 1/1.5:1). The pH was kept constant at room temperature at 6.7 ± 0.1 and 6.1 ± 0.1 at the beginning and end of the growth, respectively. The length is then significantly reduced to approximately 250 nm as the (Zn(NO_3_)_2_): (HMTA) ratio is reduced further from 0.66 to 0.25, and the pH is significantly increased at the start and end of the growth to 7.0 and 7.7, respectively. More importantly, as the HMTA fraction increased, the diameter of the ZnO NWs decreased dramatically, whereas their length increased exponentially. This is direct evidence that HMTA inhibits the radial growth of ZnO NWs while promoting axial growth. Furthermore, it was discovered that HMTA has a direct effect on the nucleation process of ZnO NWs and, thus, on their density for a given number of nucleation sites by interacting with the ZnO seed layer. Sugunan et al. [119] investigated the role of hexamine in the hydrothermal seeded growth of ZnO nanowires. Their findings show that highly anisotropic growth can be achieved with a nearly non-noticeable increase in the diameter of the resulting nanowires (during the hydrothermal growth period). According to these findings, hexamine acts as a shape-inducing molecule by selectively capping the nonpolar crystallographic planes of zincite crystals. Nanowires with typical diameters of 30 nm and lengths exceeding several microns were produced after a 24 h growth period at 60–95 °C. According to these findings and other reports in the literature, the concentration of precursors in the chemical bath can have a significant impact on the growth rate of the rod. By maintaining the precursor concentration in the bath at 1 mM, very slow but anisotropic growth was observed. Hexamine is also considered to be a nonpolar long-chain polymer that preferentially adheres to and covers all of the nonpolar faces of the ZnO crystal. The ions from the precursor can then connect to the polar (0001) face, allowing epitaxial growth along the *c*-axis. Hexamine acted as a chelating agent and determined the shape of the as-grown nanorods. The best molar ratio of the zinc source and hexamine in the precursor for the hydrothermal synthesis of ZnO nanorods was 1:1. Feng et al. [136] described their work on ZnO crystals conducted using a simple aqueous solution approach with zinc acetate-hexamethylenetetramine (HMTA) solution to understand the role of HMTA in the formation processes of ZnO nanocrystals. According to these findings, the HMTA concentration has a significant impact on the growth rate of the produced ZnO products by influencing the composition of the zinc complex species and the rate of building block transformation. They claimed that increasing the concentration of HMTA in the solution increased the average rod size from 5 to 10 µm. When heated, HMTA decomposes to form formaldehyde and ammonia, which combine with water to form OH (Equations (1)–(8)), which is discussed throughout the chemistry that drives the crystallization and creation of ZnO nanostructures. According to these findings, the concentration of HMTA influences the properties of ZnO crystals.

### 5.4. Effect of Annealing Temperature

Temperature, like other variables, has a significant impact on the formation of ZnO nanostructures. Temperature control is one way of controlling the distribution of charge carriers in the band gap region. It has been reported that when the temperature of a semiconductor increases, the energy bandgap decreases [137]. The temperature has been observed by many scientists to have a significant impact on the shape of the ZnO nanostructures. Gopal et al. [138] published a comparative study of the effect of temperature on the morphology of ZnO nanostructures. XRD and scanning electron microscopy (SEM) were used to investigate the size, shape, and arrangement of the synthesized nanoparticles. SEM was used to investigate the effect of temperature on the structural behavior of the samples before and after calcination. The average crystal size of the generated ZnO nanoparticles was estimated, and the purity of the produced ZnO nanoparticles was determined. Besides that, UV–visible spectrophotometric analysis was used to calculate the bandgap energies of the produced particles. Urgessa et al. [95] examined the lower-temperature production of well-aligned ZnO nanorods on a silicon substrate. The manufacture of ZnO nanorods on a pre-coated p-type silicon substrate is described using an aqueous solution method, which is simple, effective, and reproducible.

Ammonium hydroxide was used as the hydroxyl precursor to prepare the nanorod solution at a low temperature. By varying the seed layer solution content, the effect of the pre-substrate treatment on the diameter, orientation, and crystallinity of the as-grown ZnO nanorods was investigated. The density of the nanorods increased as the concentration of the seed layer solution increased, the preferential orientation perpendicular to the substrate improved, and the average rod diameter decreased [94]. The crystallinity and optical quality of the as-synthesized materials were confirmed by room-temperature optical spectroscopy, which showed strong UV emission and minimal deep-level emission. Guo et al. [128] investigated the hydrothermal development of well-aligned ZnO nanorod arrays and the effect of preparation conditions on morphology and alignment order. Hydrothermal growth was used to create well-aligned ZnO nanorod arrays on the surface under various conditions. Scanning electron microscopy, X-ray diffraction, and photoluminescence spectroscopy were used to investigate the effect of the preparation conditions on the deposition of the ZnO nanorods. Growth conditions, such as substrate pretreatment, growth temperature, deposition timing, and precursor concentration, have a significant impact on the morphology and alignment ordering of ZnO nanorod arrays.

Pre-treatment of substrates, which includes a dispersion of ZnO nanoparticles and subsequent annealing, not only controls rod diameter but also significantly enhances rod orientation. Although pre-coated ZnO nanoparticles play a major role in determining the rod diameter and distribution, the concentration of the precursors can be used to monitor them to some extent. The orientation of the nanorods is not significantly influenced by the growing temperature, but it has a significant impact on their aspect ratio and photoluminescence [139]. According to kinetic studies, the growth of ZnO nanorods has the following two distinct phases: rapid phase changes that occur over 60 min and produce short, wide nanorods, and a slow phase that produces long rods with a high aspect ratio. Liu et al. [140] used a hydrothermal approach to investigate the influence of pre-annealing sputtered ZnO seed layers on the formation of ZnO nanorods. A hydrothermal technique was used to fabricate oriented ZnO nanorods on ion-beam-sputtered seed layers without the use of a metal catalyst. Before the formation of ZnO nanorods, the sputtered ZnO seed layers were pre-annealed at various temperatures. The impact of pre-annealing of the seed layers on the growth rate, crystallinity, and optical characteristics of the ZnO nanorods was investigated. The produced ZnO nanorods exhibited a wurtzite structure and grew in a direction normal to the substrates along the favored (0001) orientation. The results reveal that the pretreatment conditions of the ZnO seed layer have a significant impact on the growth rate and density of ZnO nanorods [141]. Higher pretreatment temperatures improved the crystallinity and surface properties of the ZnO seed layer, resulting in a faster growth rate of the ZnO nanorods. According to the photoluminescence spectroscopy results, the UV emission becomes brighter and sharper as the annealing temperature of the ZnO seed layer increases. Table 3 shows the variation in the length, diameter, and structural parameters of the ZnO nanorods grown at different temperatures.

The effects of growing conditions on characteristics of as-synthesized ZnO nanorods grown on ZnO seed layers by ultrasonic spray pyrolysis deposited ZnO seed layers were investigated by Mosalagae et al. [49]. The objective was to determine how different chemical bath deposition conditions, such as growth time, bath temperature, and precursor concentration levels, affect the orientation and structural, optical, and vibrational properties of the resulting nanorods. Under different growth conditions, XRD confirmed the presence of hexagonal wurtzite-like ZnO nanorods with a preference for orientation along the *c*-axis and changing crystallinity. Images of uniformly oriented ZnO nanorods produced at a substantially higher bath temperature of 90 °C and a shorter development period of 120 min were obtained using scanning electron microscopy (SEM). The nanorods had an optical transmittance of 50–70%, according to UV/Vis/NIR spectrophotometer studies. Raman spectroscopy data confirmed the presence of Raman active E_2_(low) and E_2_(high) modes corresponding between 98 cm^−1^ and 478 cm^−1^ in the hexagonal ZnO phase. Manthina et al. [142] investigated the number of densities and diameter control of chemical bath-formed ZnO nanorods on FTO via forced hydrolysis of seed crystals. ZnO seed crystals were deposited on a substrate using an ethanolic Zn^2+^ precursor solution, which was subsequently immersed in an aqueous Zn^2+^ precursor solution to generate nanorods. Before depositing the seeds on commercial fluorine-doped tin oxide (FTO)/glass substrates, a force-hydrolysis procedure was used, which involved adding water and heat to the seed precursor solution. Chemical bath deposition was used to generate the ZnO nanorods from the seeds. They discovered that forced hydrolysis increased the seed crystallite size while reducing the number of seeds deposited. The numerical density of the nanorods decreased as the seed size increased, whereas the length and diameter of each rod increased. Unlike other approaches that require smoother substrates, these findings provide a simple way to control the number density of the ZnO nanorods, which is consistent with the rough FTO surface. Tlemcani et al. [86] studied the impact of ZnO seed layer deposition time and annealing on the vertical alignment of piezoelectric ZnO nanowires.

Well-aligned crystalline ZnO NWs were grown on ZnO/Au/Ti/Si substrates using a hydrothermal approach. By varying the deposition duration, radiofrequency sputtering was used to create ZnO seed layers with thicknesses ranging from 5 to 100 nm. Subsequently, the seed layers were post-annealed in the air at 400 °C. The effects of the ZnO seed layer deposition time and annealing treatment on the subsequent growth of ZnO NWs were studied using XRD, AFM, and SEM. The experimental results showed that the thickness and heat treatment of the ZnO seed layers affected the quality and growth behavior of the ZnO NWs. This work is a refinement of a previously established simple, cost-effective, and industrially scalable process flow for fabricating a high-performance nanocomposite-based stretchable nanogenerator (SNG) on a polydimethylsiloxane (PDMS) substrate. Greater performance SNGs for targeted applications of mechanical energy harvesting, such as supplying flexible and wearable electronics, may be developed because of the morphological improvement of hydrothermally produced ZnO NWs.

## 6. Fabrication of Piezoelectric Nanogenerator (ZnO-PENG)

**Figure 10:**
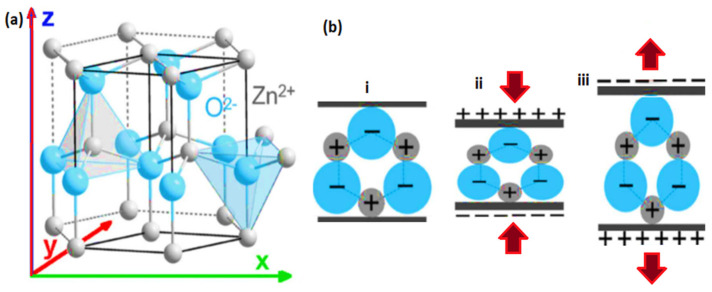
(**a**) Lattice structure of wurtzite ZnO. (**b**) Sketch of a wurtzite ZnO lattice at rest (**i**), under compressive (**ii**) and tensile (**iii**) stress, highlighting the change in the internal charge distribution of the lattice. Reprinted with permission from ref. [94]. Copyright 2019 John Wiley and Sons.

The concept of ZnO-based nanogenerators for energy harvesting is not novel [143]. However, contributions to the field of piezoelectric nanomaterials and nanogenerators have increased considerably. Piezoelectric nanogenerators are based on the piezoelectric phenomenon, which converts mechanical energy into electrical energy and vice versa. As shown in Figure 10a,b, the crystal structure of ZnO is non-centrosymmetric, with alternating layers of positive and negative ions, resulting in spontaneous polarization. Many hybrid nanogenerators have been developed since the discovery of piezoelectric nanogenerators based on ZnO nanostructures [10,117,143,144].

**Figure 11:**
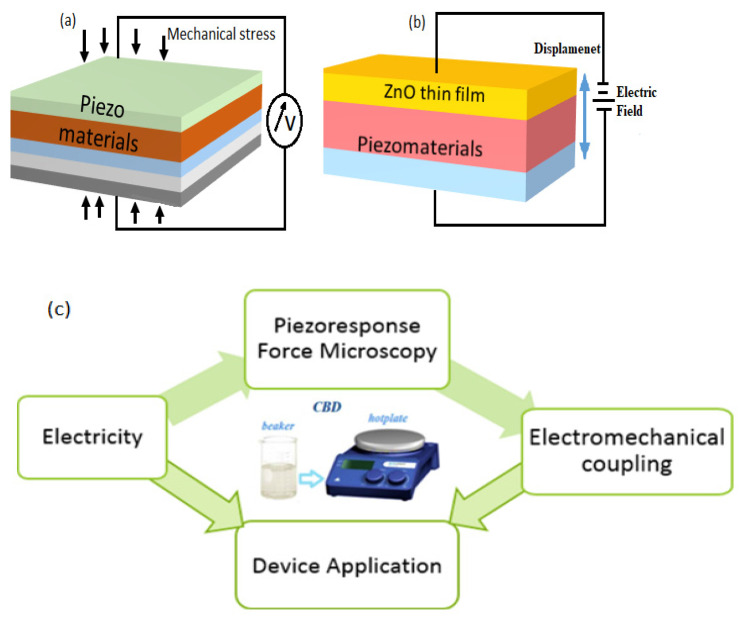
Schematic representation of (**a**) piezoelectric effect and (**b**) inverse piezoelectric effect on piezo materials and (**c**), the entire process from material’s sythesis to piezoresponse application.

An electrical potential is generated when mechanical stress is applied to the piezoelectric materials. Owing to its non-central symmetric structure, zinc oxide is a versatile II-VI metal oxide semiconductor material with anisotropic piezoelectric properties [145]. Previously, high-aspect-ratio ZnO nanostructures were synthesized by hydrothermal and CBD techniques [146,147], and these nanowires, nanorods, and nanobelts have piezoelectric capabilities (that is, they create electrical energy when mechanical stress is applied [100]. The piezoelectric property of single-crystal solids, such as ZnO, is derived from the atoms, and the asymmetric distribution of positive and negative charges begins with a unit cell and extends across the material. Figure 11 shows a schematic representation of (a) the piezoelectric effect, (b) the inverse piezoelectric effect on piezoelectric materials, and (c) the entire method and principle of piezo response measurement on materials.

Therefore, an understanding of the piezoelectric characteristics of nanomaterials as energy harvesters is required before their fabrication. Stretching a single ZnO nanorod using conductive atomic force microscopy (AFM) revealed the first observations of the converse piezoelectric effect [9]. Using piezoelectric zinc oxide nanorod (NRs) arrays, nanoscale mechanical energy was converted into electrical energy in their study. The aligned NRs were deflected in contact mode using a conductive tip [148]. Bending causes a strain field and charge separation across the nanorods, owing to the interaction between the piezoelectric and semiconducting properties of ZnO. The rectifying property of the Schottky barrier formed between the coated tip and nanorods generates an electrical current [149]. When powered by a 41 kHz ultrasonic wave, Wang et al. [147] created a vertically aligned ZnO nanogenerator that generated a unidirectional current of 0.15 nA, an open-circuit voltage of 0.7 mV, and an output power volume density of 1–4 W/cm^3^. Because the nanowires are deflected less by ultrasonic vibrations, this voltage is lower than that obtained with an atomic microscope probe. The output power density for a nanowire density of 20 µm^−2^ is ~10 pW/µm^2^ [126].

**Figure 12:**
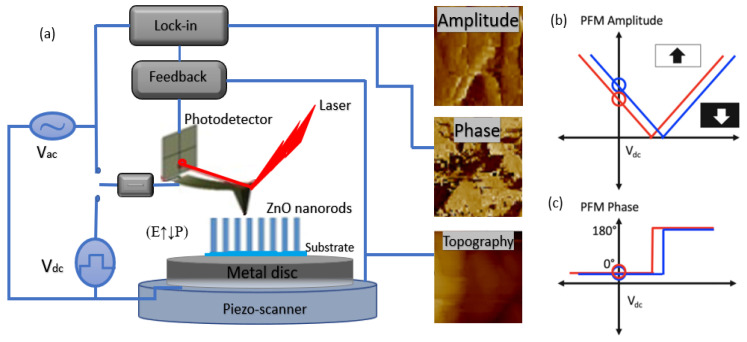
(**a**) Schematic of PFM measurement set-up with topography, phase, and amplitude images. The images show phase angle is either 0° or 180°, indicating that the polarization vector pointed either downward to the bottom electrode or upward to the samples’ surface, while (**b**,**c**) are the local hysteresis loops as a function of *V_dc_* to the ZnO NRs sample for up (red line) and down (blue line) domains. Reprinted with permission form ref. [89]. Copyright 2021 Elsevier. Reprinted with permission from ref. [152]. Copyright 2021 AIP Publishing.

The Schottky barrier formed between the microscope metal tip and nanowires generates power with a power conversion efficiency of 17–30% [150]. Hinuma et al. [151] calculated the piezoelectric potential distribution of a nanowire with a diameter of 50 nm and a length of 600 nm as 0.3 V using perturbation theory. According to the calculations, the piezoelectric potential on the nanowire surface is directly proportional to the lateral displacement of the nanowire and inversely proportional to its length, diameter, and aspect ratio. To deflect the aligned nanowires, a conductive atomic microscope with a platinum-coated silicon tip in the contact mode was used. The schematics of the PFM amplitude and phase are shown in Figure 12.

**Figure 13:**
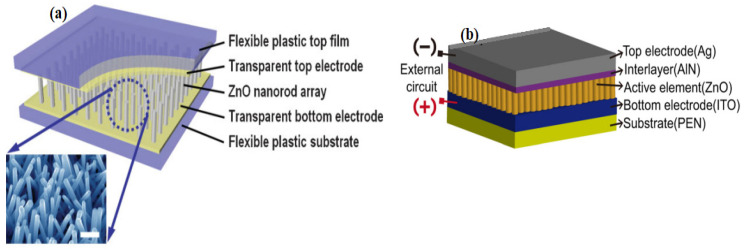
(**a**) Flexible transparent charge-generating piezoelectric nanodevices with ZnO nanorods. Inset; FESEM image of ZnO nanorod arrays sandwiched between the flexible plastic substrates. (**b**) Photographic image of the fully flexible charge-generating ZnO nanorod device with stacked ZnO/AlN-film substrates. Reprinted with permission from ref. [157]. Copyright 2009 John Wiley and Sons. Reprinted with permission from ref. [158]. Copyright 2015 AIP Publishing.

Lin et al. [153] fabricated a ZnO nanowire-based hybrid nanogenerator based on cadmium sulfide. Hydrothermal and physical vapor deposition techniques were used to produce the nanowire. Surprisingly, nanowires prepared by the physical vapor deposition method appeared to yield more voltage than nanowires prepared by the hydrothermal method. In the same year, Qin et al. [154] used a hydrothermal method with a ZnO thin-film layer as an electrode to harvest energy in a microfiber-based PENG With a power density of 20–80 mW/cm^2^, this composite structure produces an output voltage of 1–3 mV and a current of 4 nA. In contrast, Zhao et al. [155] calculated the substrate’s (negative) contribution to ZnO piezoelectric energy harvesting, which resulted in a 50% reduction in the effective piezoelectric coefficient. The Schottky barrier produced between gold and ZnO, according to Wang et al. [156], has significantly improved with a detectable voltage output of approximately 10 mV. They hypothesized that the stretched side of the nanorod had a positive potential, resulting in a reverse bias with the Schottky junction, which blocked any current from flowing to filter the polarization. The connection was forward-biased as the tip reached the compressed, negatively polarized side of the rod, allowing the current to pass through the screen for polarization. Flexible transparent charge-generating piezoelectric nanodevices with ZnO nanorods and FESEM images of ZnO nanorod arrays sandwiched between flexible plastic substrates. As shown in Figure 13a,b, an image of flexible charge-generating nanodevices with the top and the bottom film substrate as electrodes. Figure 13b. the structure model of epitaxially grown ZnO nanorod arrays sandwiched between flexible indium-tin-oxide/polyethylene-naphthalene (ITO/PEN) substrates.

**Figure 14:**
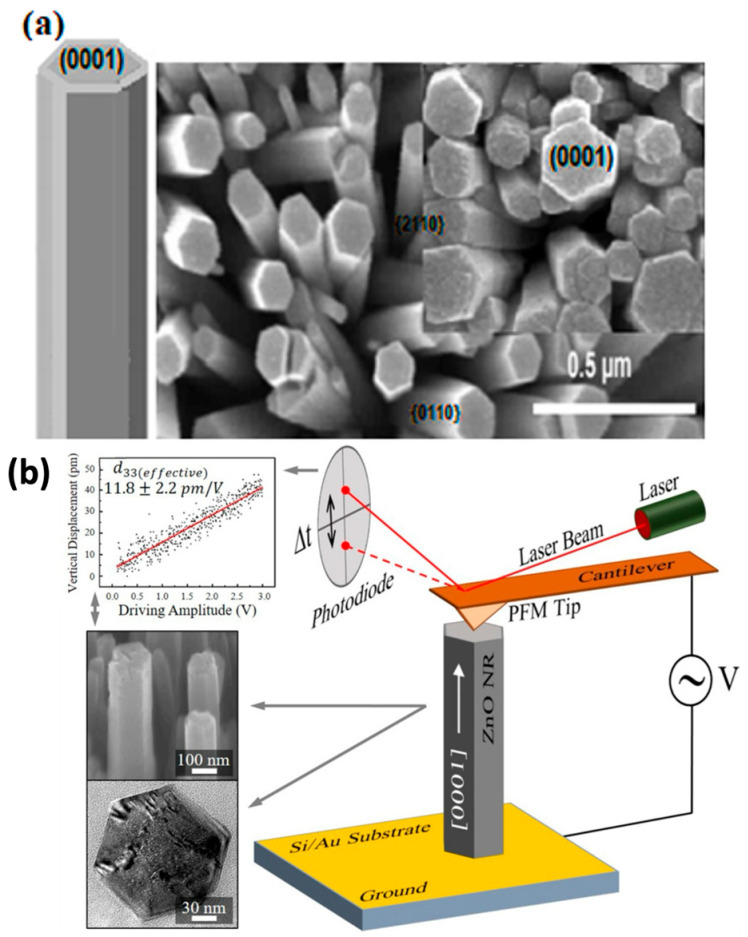
(**a**) FE-SEM image of ZnO nanorod arrays grown on a seeded substrate, with hexagonal (0001) surface. (**b**) PFM model with precise cantilever deflections on the nanorod. Reprinted with permission from ref. [89]. Copyright 2021 Elsevier. Reprinted with permission from ref. [146]. Copyright 2015 Elsevier.

**Figure 15:**
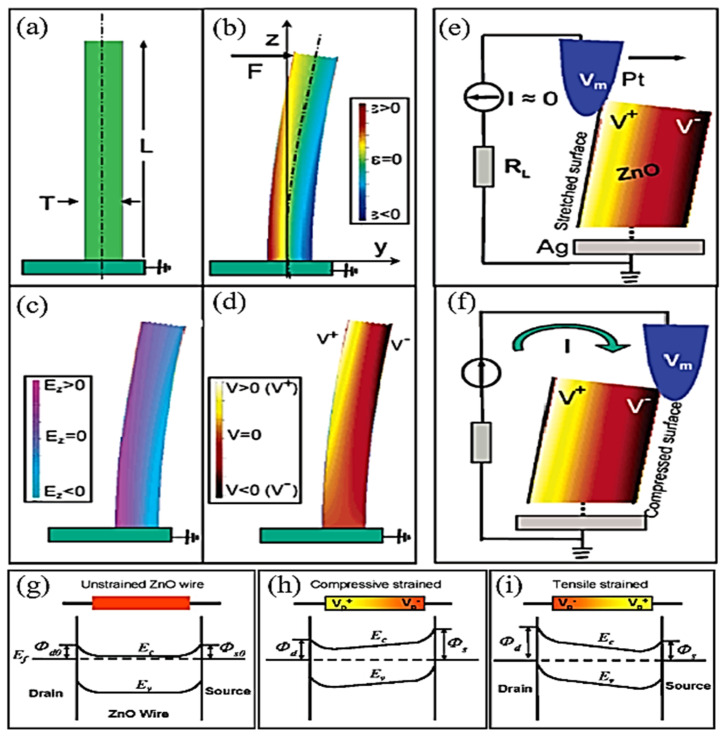
(**a**–**d**) A unique coupling of piezoelectric and semiconducting properties for the power-generating processes of a ZnO nanowire with thickness and length. (**e**,**f**) Schematic contact field between the conductive AFM tip and single ZnO, showing reverse- and forward-biased Schottky rectifying behavior. Figure (**g**–**i**) The fields developing within horizontal ZnO NWs and near the Schottky contacts for unstrained, compressed, and stretched NWs Reprinted with permission from ref. [143]. Copyright 2006 American Chemical Society. Reprinted with permission from ref. [160]. Copyright 2018 Elsevier.

Figure 14 shows ZnO nanorods with hexagonal (0001) facets grown on a seeded substrate. The PFM model illustrates the cantilever deflections on the nanorod. During scanning with the AFM tip, a characteristic DC voltage is applied, creating an open-circuit voltage [159], as shown in Figure 15.

## 7. A Facile Synthesis of ZnO Nanorods for PENG Performance Enhancement

**Figure 16:**
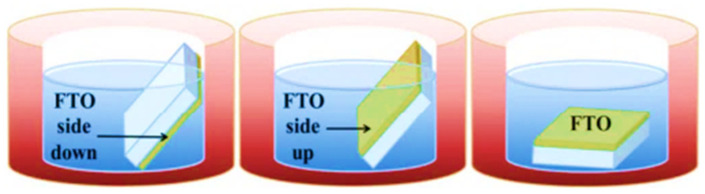
Schematic illustration of the 3-set positions of the FTO-coated side substrate. Reprinted from ref. [160].

The performance of a piezoelectric nanogenerator (PENG) composed of ZnO nanowires and nanorods is primarily determined by the initial controlled synthesis and fabrication conditions of the device, as well as the structural, mechanical, and electrical properties. Different morphologies of the same material, such as nanowires and nanorods, can be synthesized by altering their growth parameters. Previous studies have focused on optimizing the parameters, resulting in a variety of zinc oxide nanostructures that were classified based on their dimensionalities. As previously demonstrated, different morphologies have a significant impact on the performance of piezoelectric nanogenerators, depending on their initial growth conditions. Sutapa et al. [160] investigated the influence of hydrothermal variables and substrate position on the growth process and morphology of TiO_2_ nanostructures grown on an FTO-coated glass substrate. Their work largely depended on the synthesis conditions and process parameters for the growth of nanostructures. In summary, research shows that the growth of TiO_2_ nanostructures is dependent on these variables and results in distinct morphologies. They conducted a systematic study to better understand the parameters involved and substrate orientation (horizontal and tilted at an angle), as shown in Figure 16.

**Figure 17:**
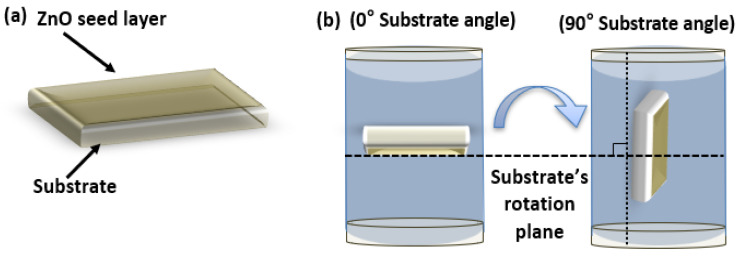
(**a**,**b**) ZnO nanorods growth prepared on the substrate for 0° and 90° substrate angles. Reprinted from ref. [161].

Puenisara et al. [161] use the aqueous solution method to investigate the effect of precursor concentrations and substrate angles on ZnO-NR morphology growth. ZnO nanorods were synthesized on a silicon substrate with a seed zinc layer, and the morphology of the nanorods was studied at substrate angles of 0° and 90°. According to the SEM micrographs, the growth of ZnO nanorods with a 0° substrate angle is smaller than that with a 90°substrate angle, as shown in Figure 17.

The findings also show that the substrate angle affects the external force acting on ZnO particles deposited on the substrate. When ZnO was placed at a 90° angle on the substrate, lateral growth outpaced longitudinal growth. Samples grown in such a well-controlled position can allow precise estimation of the vertical piezoelectric strain coefficient during the sample/tip interaction in the PFM contact mode. In another study, Xu et al. [162] successfully integrated ZnO nanowires under a strain of 0.19% to produce a peak voltage of 1.26 V and a maximum current of 28.8 nA. The growth mechanism of ZnO nanostructures for ultra-high piezoelectric coefficients (*d*_33_) was investigated using the idea of Ghosh et al. [8]. Molarity, temperature, growth period, and seed layer are some of the main regulatory parameters. Mufti et al. [163] investigated the structural and morphological properties of ZnO nanorods produced by varying the precursor ratio on a stainless-steel substrate, as well as the effect on piezoelectric nanogenerator performance. ZnO nanorods were produced using a modified hydrothermal technique (HMT) by adjusting the molar ratio of zinc nitrate tetrahydrate (ZNT) to hexamethylenetetramine (HT). The performance of the piezoelectric nanogenerator was assessed by monitoring its voltage and current while applying an external force to the device. By increasing the ZNT/HMT ratio, the average length of the ZnO nanorods increased, whereas the average diameter decreased. The current and voltage of the piezoelectric nanogenerator were enhanced when the zinc nitrate ratio was increased. These findings suggest that the ratio of the ZNT to HMT precursors is critical for the performance of piezoelectric nanogenerators. Table 4 shows that this study obtained an excellent piezoelectric coefficient (*d*_33_) of 44.33 pm/V for vertically aligned ZnO nanorod structures, which is the highest known *d*_33_ value for any type of ZnO nanostructure. On the other hand, the XRD analysis confirmed the wurtzite character of this nanorod structure, with [0001] being the preferred growth direction. Temperature-induced I/V characterization was also performed to determine the semiconducting properties of the nanorods. Zhu et al. [164] synthesized vertically aligned piezoelectric ZnO nanowires to demonstrate a new type of integrated nanogenerator. With a maximum power density of 0.78 W/cm^3^, the peak open-circuit voltage and short-circuit current achieved high values of 58 V and 134 µA, respectively. Flexible piezoelectric nanomaterials have attracted considerable attention owing to their high phase content, which results from their crystallization and simple processing conditions. Electroactive polymers, such as polyvinylidene fluoride (PVDF) and its copolymer, (polyvinylidene fluoride-tetrafluoroethylene) (PVDF-TrFe), have received particular attention among piezoelectric materials. Hu et al. [165] improved the performance of nanogenerators by using pretreatment techniques such as oxygen plasma, annealing air, and surface passivation with different polymers on the produced ZnO nanowire films. The output voltage of the nanogenerator exceeded 20 V, and its output current exceeded 6 µA. According to theoretical simulation [166], the piezoelectric potential of a nanowire is proportional to its deformation in the elastic linear mechanical region. The rods in the integrated nanogenerator were connected in parallel between the two electrodes. As the external strain increases, so does the deformation, and the power output voltage increases accordingly [167].

The frequency at which external strain is applied also influences the magnitude of the output voltage [146]. This output can be utilized as a power source for nanodevices, but further research is needed for effective utilization. However, it was found that free charge carriers occur in piezoelectric materials owing to surface desorption and native defects [32,170,171].

Lu et al. [172] investigated the effect of free charge carriers on the piezoelectric potential of ZnO nanorods. Improved intrinsic features, such as surface passivation, thermal annealing, and oxygen plasma, can also reduce the carrier concentration. The piezoelectric constant (*d_33_*) was used to estimate the performance of ZnO-PENG in the current digital era. Atomic force microscopy is used to calibrate the parameters. Piezoresponse force microscopy (PFM) [173,174] is one of the most frequently used methods for characterizing nanostructures and measuring the produced potential. The inverse piezoelectric characteristics of many materials have been investigated using this method [35]. The experimental settings for measuring the piezoelectric characteristics of the ZnO nanowires using PFM were consistent with procedures described elsewhere [175].

## 8. Conclusions, Recommendations, and Future Prospects

This review primarily focuses on the synthesis and characterization of ZnO nanomaterials. Research and applications of nanowires and nanorods (NWs/NRs) are currently gaining attention because of their enhanced properties at the nanoscale and their ability to be integrated into many electronic devices. Piezoelectric nanomaterials have a competitive advantage over other bulk materials and can withstand numerous integration routes. The effect of physicochemical synthesis parameters on ZnO nanomaterials with varying structural and morphological textures is investigated in this review. Various AFM micrographs and FESEM images show clear evidence of the relationship between the initial growth of the seed layers and subsequent nucleation of the nanostructures on different substrates. In our preliminary study [89], we investigated the effect of the seed layer deposited on different substrates (silicon, ITO, and glass) and their subsequent influence on the growth orientation of ZnO NRs. Generally, our findings reveal that well-aligned ZnO nanorods are more stable because of the random orientation of crystalline grains on the seeded substrates. As a result, the diversity of nanorod orientation is mutually intersected, thereby forming an interconnected network. Therefore, it is suggested that by controlling the growth parameters during the synthesis, the morphology and size of the nanorods can be tuned for piezoelectric applications. This piezoelectric effect in ZnO nanorods is very responsive to external detection devices such as sensors and actuators or in tunneling, probes to measure cantilever deflection, which can be utilized to initiate an electromechanical response in individual ZnO nanorods via electrical excitation and detection. The piezoelectric coefficient factor that determines the electromechanical performance of NWs and NRs generators is strongly dependent on the controlled growth and stable crystal structure of nanomaterials. This phenomenon can be comprehensively investigated through PFM and AFM. Based on this technique, the piezoresponse properties of NWs and NRs were investigated and compared according to the coefficient factor (*d_33_*).

Notably, this review highlights various ZnO hybrid nanomaterials for PENG applications in recent years, suitable for improving the energy harvesting capabilities of ZnO nanogenerators, along with other modification techniques. Further discussions on some published works on ZnO-PENG nanogenerators and their fabrication methods are summarized in Table 5. for a variety of applications. However, further research to improve the structural, mechanical, and electronic properties of new materials and composites for device fabrication is an effective way to achieve the optimum performance of ZnO piezoelectric generators with output potential for piezoelectric energy harvesting capabilities. However, the major constraint in this direction is the understanding of ZnO-PENG piezoelectric energy-harvesting devices with complete rectifying voltage output, long-term performance, and life cycle. These results demonstrate that the greater efficiency in terms of device performance is linked to the electromechanical properties of the nanostructures. Additionally, some results for improving piezoelectric performance through the substrate position have been presented from the perspective of micro/nanostructures, which include size, crystal structure, orientation, and defects. Despite the advantages of various piezoelectric materials, nanostructured composites and heterostructures with various piezo/ferroelectric properties can be used to achieve additional advantages, and their applications in nanogenerators have grown rapidly.

Finally, integrating these nanostructures into self-powered devices can effectively produce electrical contacts with low maintenance requirements. The development of hybrid nano energy harvesters capable of converting other forms of energy into electricity will be the focus of future research. In conclusion, it is expected that this work will help researchers explore the exotic properties of ZnO nanostructured materials, especially for energy and environmental applications. This review covers the material structure, synthesis, characterization, and performance enhancement techniques. More efforts should be made to investigate cost-effective methods and other materials with enhanced piezo-electromechanical response properties.

## Figures and Tables

**Table 1:** Growth deposited parameters of sputtered ZnO thin film on substrates, with different surface roughness values and thickness.

Target	Sputtered Material	Substrate	Temp. (°C)	Power (W)	Pressure (torr)	Ar: O_2_ Flow [sccm]	Deposition Time [min]	Thickness (nm)	Surface Roughness (nm)	Growth	Ref
ZnO	ZnO film	Si (100)	RT	200 W	3 × 10^−2^	1/10	variable	20–1000 nm	0.55–3.80 nm	NWs	[62]
ZnO	ZnO film	Si (100)	RT	100 W	controlled	40–80	variable	10–40 nm	variable	NRs	[63]
ZnO	ZnO film	Si (100)	RT	100 W	1 × 10^−2^	variable	variable	20–320 nm	0.55–3.80 nm	NRs	[64]
ZnO	ZnO film	Si/Al/SiO_2_	RT	70 W	1 × 10^−4^	variable	60 min	0.252–0.372 µm	variable	Piezo-film	[65]
ZnO	ZnO film	quartz	variable	variable	2 × 10^−4^	variable	30 min	variable	variable	Thin film	[66]
ZnO	ZnO film	Si (100)	600	80 W	1.0 × 10^−5^	variable	variable	variable	variable	NWs/NRs	[67]
ZnO	ZnO film	Si (100)	RT	120 W	controlled	variable	variable	80–200 nm	variable	NRs	[68]

**Table 2:** Illustration of selected experimental data and deposition-growth parameters for ZnO seed layer and ZnO nanorod arrays synthesized by chemical bath deposition and hydrothermal methods.

Molar Ratio/ Concentrations	Temp. [°C]	Growth Time	pH	Substrate	Seed Layer/ Seedless	NRs Diameter	NRs Height	Ref
ZN: HMTA; 1:1; 0.05 M	95	2h		silicon	RF-sputtered	30–72 nm	967–1052 nm	[64]
ZN: HMTA; 1:1; 50 mM	90	1 h	NRs	silicon	sputtered ZnO seed	50–150 nm	542–695 nm	[68]
ZN: NH_4_OH: PVD 1:1; 64 Mm: 2.56 M	65	90 min	NRs	silicon	sol-gel solution	50–470 nm	(variable)	[95]
ZN: HMTA; 1: 1; 0.01, 0.001 M	95	10 h	NRs	FTO, Si, single crystal sapphire	seedless/seed	200–10 nm	10–2 µm	[120]
ZN: HMTA; 1: 1, 0.025 M	90	1.5 h	NRs	silicon	spin-coated ZnO nanocrystals	40–80 nm	1.5–2 µm	[127]
ZN: HMTA; 1:1, 0.1 M	95	20 min–2 h	NRs	ITO/glass	ZN or ZA	30–250 nm	0.25–4 µm	[128]
ZN: HMTA: PEI; 1:1; 0.025–0.05 M, 0.025 M and 0.005–0.025 M	85	6 h	NRs	glass	sputtered ZnO seed	4–300 nm (variable)	(variable)	[129]
ZN: HMTA; 1: 1; 10–50 mM	95	5 h	NRs	silicon	sputtered seed ZnO	50–160 nm	2600–2500 nm (variable)	[51]
ZnS: NH_4_Cl; 1:1; 10 mM: 300 mM	60	6 h	11	glass slides	sol-gel solution	100 nm (average)	1.5 µm (average)	[130]

**Table 3:** Variation of the length, diameter, and structural parameters of the grown ZnO nanorods at different temperatures. Adapted from ref. [49].

Growth Temp. (°C) Range	Average Length of the Nanorods (nm)	Average Diameter of the Nanorods (nm)	Crystalline Size/Density of NRs (nm)/µm^2^	Ref.
60–90	400 ± 26 700 ± 64	129 ± 17 143 ± 25	61.3–67.5	[49]
70–80	570 ± 18 480 ± 40	70 ± 30 90 ± 20	35.0–54.0	[86]
70–90	3.0–10	0.2–1.4	20.1–0.21	[142]

**Table 4:** Piezoelectric coefficient *d*_33_ and characterization of ZnO nanostructures using PFM analysis.

Material	Characterization	Piezoelectric *d*_33_ (pm/V)	Ref.
ZnO NRs	PFM on NRs	*d*_33_ = 49.7 pm/V	[9]
ZnO NRs	PFM on NRs	*d*_33_ = 4.41 ± 1.73 pm/V	[35]
ZnO NRs	PFM on NRs	*d*_33_ = 11.8 pm/V	[146]
ZnO NBs	PFM on NBs	*d*_33_ = 14.3–26.7 pm/V	[155]
ZnO NRs	PFM on NRs	*d*_33_ = 0.4–9.5 pm/V	[168]
ZnO NRs/NWs	PFM on NRs/NWs	*d_33_* = 7.01 ± 0.33 pm/V and 2.63 ± 0.49 pm/V	[169]

**Table 5:** Summary of ZnO-PENG method of fabrication and integration for different applications.

Material	Nanogenerator	Method of Integration	Output	Remarks	Ref.
ZnO based NG	PENG, vertical ZnO nanowire arrays, ultrasonic wave driven	high temp. growth of ZnO on different substrates	NG ~5 mV	PENG improved power generation ZnO nanowires grown on various substrates	[111]
ZnO based NG	ZnO-PENG	vertically aligned ZnO NW on the substrate	Output voltage detected.	powering nanodevice with ZnO nanowire-based generator.	[143]
ZnO NRs-NG	PENG (ZnO nanorod)	ZnO-PENG by controlling the diameter and height of NRs	~10.4 nA/cm^2^	ZnO NRAs exhibited a relatively regular and high output current due to the efficient bending under a low external force of 0.98 N.	[144]
ZnO hybrid PENG	PENG Vertically aligned ZnO NW array	PN-hetero junction of QD and NG with ITO (top) and Au (bottom) electrodes	NG-AC 22–45 nA 1.5–6 mV under 50Hz sound wave	solar + sound energy harvesting NG	[176]
ZnO NWs-NG	PENG Vertically aligned ZnO NW array	polycrystalline ZnO PENG combining layers fabricated at RT by plasma-assisted deposition (PLD) with supported organic nanowires.	NG ~170 mV	hybrid nanostructured PENG with core-shell, formed by a single-crystalline ZnO, combined with the mechanical properties of ZnO layer	[177]

## Data Availability

This study did not report any data.
