# Correction: Abubakar et al. Controlled Growth of Semiconducting ZnO Nanorods for Piezoelectric Energy Harvesting-Based Nanogenerators. *Nanomaterials* 2023, *13*, 1025

**DOI:** 10.3390/nano15191491

**Published:** 2025-09-29

**Authors:** Shamsu Abubakar, Sin Tee Tan, Josephine Ying Chyi Liew, Zainal Abidin Talib, Ramsundar Sivasubramanian, Chockalingam Aravind Vaithilingam, Sridhar Sripadmanabhan Indira, Won-Chun Oh, Rikson Siburian, Suresh Sagadevan, Suriati Paiman

**Affiliations:** 1 Department of Physics, Universiti Putra Malaysia, Serdang 43400, Selangor, Malaysia; shamsuabubakar05@gmail.com (S.A.);; 2 Department of Physics, Yobe State University, Damaturu P.M.B. 1144, Yobe State, Nigeria; 3 Department of Physics, College of Natural Science, Jeonbuk National University, 567 Baekje-daero, Deokjin-gu, Jeonju-si 54896, Jeollabuk-do, Republic of Korea; 4 Faculty of Innovation and Technology, Taylor’s University Malaysia, No. 1, Jalan Taylor’s, Subang Jaya 47500, Selangor, Malaysia; 5 Department of Advanced Materials Science and Engineering, Hanseo University, Seosan-si 356-706, Chungnam, Republic of Korea; 6 Department of Chemistry, Faculty of Mathematics and Natural Sciences, Universitas Sumatera Utara, Padang Bulan, Medan 20155, Indonesia; 7 Nanotechnology & Catalysis Research Centre, Universiti Malaya, Kuala Lumpur 50603, Malaysia; 8 Functional Nanotechnology Devices Laboratory (FNDL), Institute of Nanoscience and Nanotechnology, Universiti Putra Malaysia, Serdang 43400, Selangor, Malaysia

In the original publication [1], reference [159], “Khan, A.; Edberg, J.; Nur, O.; Willander, M. A novel investigation on carbon nanotube/ZnO, Ag/ZnO and Ag/carbon nanotube/ZnO nanowires junctions for harvesting piezoelectric potential on textile. *J. Appl. Phys.* **2014**, *116*, 034505”, has been retracted and removed. With this correction, the order of some references has been adjusted accordingly. The affected content in Section 6, Paragraph 6 has been updated and should read as follows:

Figure 14 shows ZnO nanorods with hexagonal (0001) facets grown on a seeded substrate. The PFM model illustrates the cantilever deflections on the nanorod. During scanning with the AFM tip, a characteristic DC voltage is applied, creating an open-circuit voltage [159], as shown in Figure 15.

In addition, Figure 14 and its caption have been updated accordingly to reflect this change. The correct Figure 14 and its caption appear below.

The authors state that the scientific conclusions are unaffected. This correction was approved by the Academic Editor. The original publication has also been updated.

## Figures and Tables

**Figure 14:**
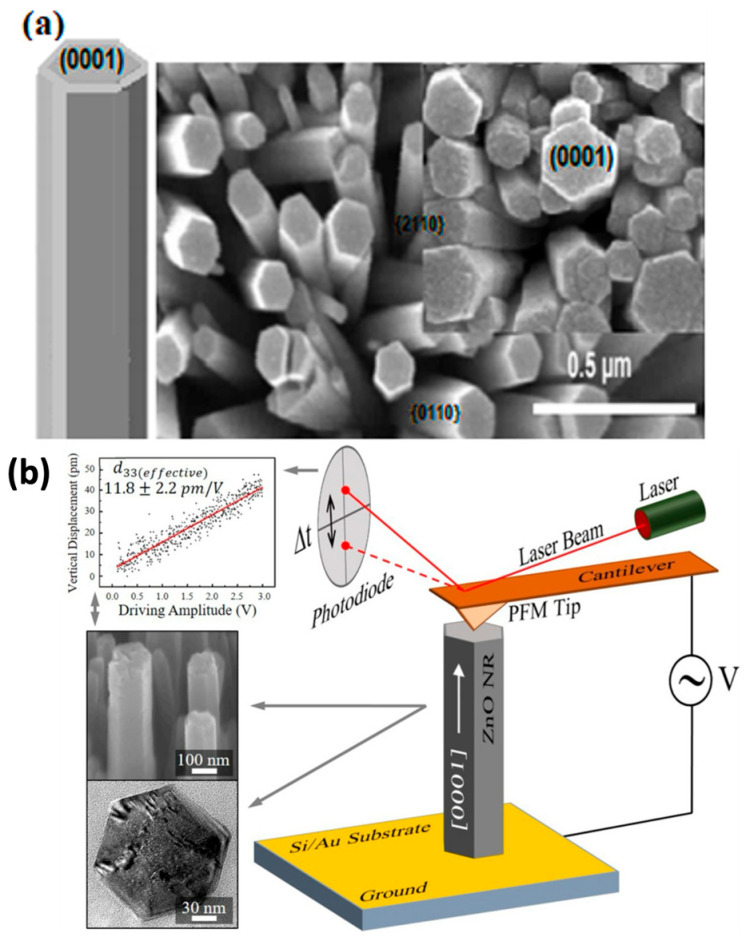
(**a**) FE-SEM image of ZnO nanorod arrays grown on a seeded substrate, with hexagonal (0001) surface. (**b**) PFM model with precise cantilever deflections on the nanorod. Reprinted with permission from ref. [89]. Copyright 2021 Elsevier. Reprinted with permission from ref. [146]. Copyright 2015 Elsevier.
